# Best practices for evaluating single nucleotide variant calling methods for microbial genomics

**DOI:** 10.3389/fgene.2015.00235

**Published:** 2015-07-07

**Authors:** Nathan D. Olson, Steven P. Lund, Rebecca E. Colman, Jeffrey T. Foster, Jason W. Sahl, James M. Schupp, Paul Keim, Jayne B. Morrow, Marc L. Salit, Justin M. Zook

**Affiliations:** ^1^Biosystems and Biomaterials Division, Material Measurement Laboratory, National Institute of Standards and Technology, Gaithersburg, MD, USA; ^2^Statistical Engineering Division, Information Technology Laboratory, National Institute of Standards and Technology, Gaithersburg, MD, USA; ^3^Division of Pathogen Genomics, Translational Genomics Research Institute, Flagstaff, AZ, USA; ^4^Center for Microbial Genetics and Genomics, Northern Arizona University, Flagstaff, AZ, USA; ^5^Department of Bioengineering, Stanford University, Stanford, CA, USA

**Keywords:** next-generation sequencing, variant calling, single nucleotide variants, indel, performance metrics

## Abstract

Innovations in sequencing technologies have allowed biologists to make incredible advances in understanding biological systems. As experience grows, researchers increasingly recognize that analyzing the wealth of data provided by these new sequencing platforms requires careful attention to detail for robust results. Thus far, much of the scientific Communit’s focus for use in bacterial genomics has been on evaluating genome assembly algorithms and rigorously validating assembly program performance. Missing, however, is a focus on critical evaluation of variant callers for these genomes. Variant calling is essential for comparative genomics as it yields insights into nucleotide-level organismal differences. Variant calling is a multistep process with a host of potential error sources that may lead to incorrect variant calls. Identifying and resolving these incorrect calls is critical for bacterial genomics to advance. The goal of this review is to provide guidance on validating algorithms and pipelines used in variant calling for bacterial genomics. First, we will provide an overview of the variant calling procedures and the potential sources of error associated with the methods. We will then identify appropriate datasets for use in evaluating algorithms and describe statistical methods for evaluating algorithm performance. As variant calling moves from basic research to the applied setting, standardized methods for performance evaluation and reporting are required; it is our hope that this review provides the groundwork for the development of these standards.

## Introduction

Next-generation sequencing (NGS) has transformed microbiology, making genomic analyses possible for a broad range of species. However, converting millions of sequencing reads per sample into meaningful data is not trivial, and genome assembly, sequence alignment, and variant calling can all have substantial effects on results. Considerable effort has been spent addressing these issues in human genomes, with a primary goal of finding genomic variations linked to human disease. Variant calling from microbial genomes presents additional challenges, such as reference genome selection, presence of rare variants within a culture, and use of *de novo* genome assemblies for variant calling. Due to the diversity of methods used to call variants from microbial genomes, optimization of bioinformatics methods for specific organisms and/or experiments is frequently required.

Variant calling can include single nucleotide polymorphisms (SNPs), insertions and deletions (indels), and/or structural variants. Here, we focus on SNPs and indels. Both SNP and indel calling methods identify genome positions with polymorphisms relative to a reference (for review, see Nielsen et al., 2011). SNP and indel calling is achieved by either mapping reads directly to the reference genome or generating a *de novo* genome assembly from the reads and subsequently comparing the assembly to the reference genome.

For SNP discovery, using raw reads can provide greater resolution than using a genome assembly. With raw reads, both the depth of coverage as well as the proportion of mixed alleles can be quantified, in contrast to creating an assembly, in which all coverage at a given locus is collapsed into a single base call. When the raw reads are available they can be mapped back to the assembly to obtain the coverage and allelic proportions. However, lack of a closed reference genome may cause biases in allelic proportions due to mapping errors. Updates to the sequence alignment format (SAM/BAM) to accommodate storing *de novo* sequence alignments can resolve this issue (Cock et al., 2015). For reference-based SNP discovery, reads can fail to align to regions of high divergence (Bertels et al., 2014) if the short read aligner is too stringent. This can be resolved with some assembly-based SNP discovery methods, as regions can be aligned from more divergent sequences, allowing for the characterization of SNPs even from different bacterial species within the same genus (Sahl et al., 2013).

Single nucleotide polymorphism identification is an important method for bacterial comparative genomics. SNP-based analyses have been used for outbreak attribution (Hendriksen et al., 2011), phylogeography (Keim and Wagner, 2009), and genome-wide association studies (GWAS; Nelson et al., 2014); SNPs have also been used extensively in human GWAS applications (Cantor et al., 2010). Multiple types of error, which must be explored and accounted for to understand the evolution and relatedness of bacterial genomes, affect SNP-based bacterial comparative genomics.

Both similarities and differences exist between human and microbial variant calling. The human reference genome is ∼1000 times larger than an individual microbial genome, and humans are diploid whereas microbial genomes are generally haploid. Therefore, the assumptions and statistics used in human variant callers often are not optimal for microbial genomes. The smaller number of mutations in a bacterial genome means that some machine learning methods used to filter potential false positives (FP) in humans will not work well for microbial genomes, and different filtering thresholds are often needed depending on the microbial sequencing application. In addition, some microbial genomes have high mutation rates, so that they may be heterogeneous, with only a small fraction of cells containing a mutation. In this case, they are more similar to somatic variant calling in human cancer cells. All of these differences mean that validating variant callers for microbial sequencing should be done even if the same variant caller has been validated for human sequencing.

Selecting the optimal variant calling method can depend on the application, organism, and sequencing data (Koboldt et al., 2009). Currently, there are no widely accepted guidelines for evaluating SNP and indel calling methods. The lack of guidelines has resulted in a diverse, inconsistent, and difficult-to-interpret body of literature on SNP and indel calling method performance. To help address this issue, we set out to provide general guidelines for evaluating SNP and indel calling methods. We briefly discuss SNP and indel calling procedures from NGS data and describe the associated errors. In order to develop guidelines for SNP and indel calling method evaluation, we discuss and identify appropriate data for use in evaluation, present statistical methods for evaluation, and methods for comparing variant call sets.

## SNP and Indel Calling Procedure

In order to draw meaningful conclusions from evaluation of variant calling methods, the process used to identify the variants must first be understood. This measurement process includes sample processing, sequencing, mapping or *de novo* assembly, followed by variant calling (Altmann et al., 2012, Figure 1).

**FIGURE 1:**
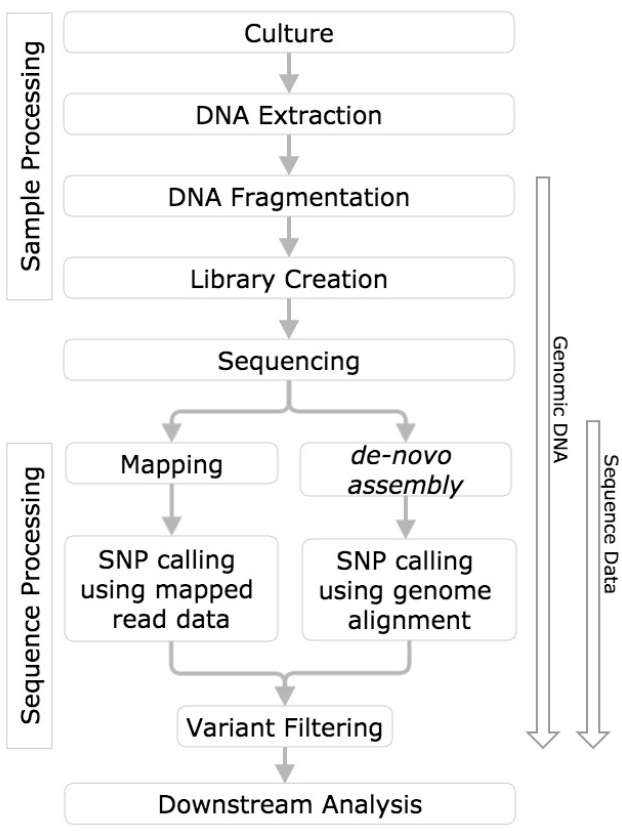
**SNP calling workflow diagram.** Horizontal boxes represent steps in the workflow and arrows to the left indicate steps in the workflow challenged with reference genomic DNA, and sequence data.

### Sample Processing and Sequencing

DNA is extracted from a bacterial culture, purified, and during library preparation, adapters and unique barcodes (indices) are added prior to sequencing.

The resulting library or pooled libraries are sequenced (for reviews of NGS platforms see Metzker, 2010; Pabinger et al., 2013).

Base calls are automatically generated by the sequencing platform. For each base call, algorithms assign a base quality score (BQS), which is intended to reflect the probability that the base was called correctly. BQS values are provided on the phred scale (1–60), as the –10 logP, where P is the probability of an incorrect base call (Ewing and Green, 1998; Pavlopoulos et al., 2013). For example, a BQS of 20 indicates a 1 in 100 chance the base was called incorrectly. BQS commonly inform variant calling software when assigning variant quality scores (also typically expressed on a phred scale).

### Sequence Processing and SNP Calling

After the raw reads are generated by the sequencing platform, reads can be mapped to a reference genome, *de novo* assembled, or mapped and assembled in one simultaneous step (Zhong et al., 2015). SNPs and indels relative to a reference are then identified by comparison to a reference genome. Commonly used read mapping and *de novo* assembly based approaches are discussed in this review.

#### SNP Calling Following Read Alignment to a Reference

***Mapping reads to a reference***

To map reads to a reference genome, the location of each read, relative to the reference genome, is predicted. To assess the performance of short read mapping tools, studies have evaluated different algorithms’ performance of short read alignment tools (e.g., Fonseca et al., 2012; Schbath et al., 2012; Hatem et al., 2013; Nagarajan and Pop, 2013). Reads are initially mapped and then a series of steps including duplicate read removal, BQS recalibration, and indel realignment are performed to refine the read alignments prior to variant calling.

Most mapping programs assign phred scale mapping quality scores to indicate the confidence that the reads are accurately placed in relation to the reference (Gallagher and Desjardins, 2008). Mapping quality scores generated by different mapping algorithms are generally not comparable to each other, but in general, lower mapping quality scores are assigned to shorter reads as well as reads mapping to multiple regions genome (e.g., repeat regions).

The resulting mapped file, usually the binary version (BAM) of a Sequence Alignment/Map (SAM) file, then undergoes additional processing to reduce variant calling errors. These include duplicate read removal, BQS recalibration, and realignment around indels. Duplicate reads originating from the DNA sequence can arise as artifacts of the PCR step in the sequencing library preparation process, or when the same fragment is read twice (i.e., optical duplicate). As such, duplicates can lead to an artificial increase in bases supporting variant calls, leading to an erroneous increase in variant call confidence, and should therefore be removed either before or after the mapping step (DePristo et al., 2011). For newer low cycle and “PCR-free” library preparation methods, read duplication is less likely to occur.

Recalibrating BQS can help improve quality score accuracy and thus, the accuracy of called variants (DePristo et al., 2011; Zook et al., 2012). To recalibrate, the sequencing error rate for positions in the reference genome that are known with high confidence are compared to the sequencing platform assigned BQS. The BQS for the entire data set are recalibrated based on differences in the assigned BQS and the observed base call error rates for the known positions.

Indels and other structural variants can lead to incorrect sequence read mapping, causing false negative (FN) and positive SNP and indel calls (Alkan et al., 2011). Therefore, algorithms have been developed to increase the accuracy of read mapping in these regions through read realignment (e.g., GATK IndelRealigner) or local *de novo* assembly (e.g., GATK HaplotypeCaller), in turn reducing the number of incorrect variant calls in these portions of the genome (Homer and Nelson, 2010; DePristo et al., 2011).

***Calling SNPs using mapped read data***

Finally, variant calling algorithms compare mapped reads to the reference genome and identify potential variants. SNP and indel calling algorithms vary in their approach to identifying candidate variants (Altmann et al., 2012). Basic algorithms identify variants based the on the number of high confidence base calls that disagree with the reference bases for the genome position of interest. More sophisticated algorithms commonly use Bayesian, likelihood, or machine learning statistical methods that factor parameters, such as base and mapping quality scores, to identify candidate variants. See Pabinger et al. (2013) for a review of different variant calling algorithms. The presumptive SNPs and indels identified by the variant caller can be filtered using a number of parameters associated with systematic errors discussed in the next section, thereby reducing the number of FP variant calls, but risking an increase in FN calls.

#### SNP Calling Using *de novo* Assemblies

***De novo genome assembly***

Many applications in comparative genomics, such as pan-genome comparisons, operon structure determination, or genome synteny in a population, require *de novo* genome assembly. For short read *de novo* assembly, *de Bruijn* graph methods are typically used (Chaisson and Pevzner, 2008), although overlap layout consensus can also be used effectively (Loman et al., 2012). In both methods, using paired-end and/or mate pairs can increase the contiguity of an assembly by facilitating the generation of genomic scaffolds, which link contigs based on insert length. For graph-based methods, the choice of *k*-mer size (base string length) can affect the contiguity and/or completeness of an assembly (Chikhi and Medvedev, 2014). For some *de novo* assembly methods, such as Velvet (Zerbino and Birney, 2008), a single *k*-mer value is frequently chosen. For newer methods, such as SPAdes (Bankevich et al., 2012) or IDBA (Peng et al., 2010), assemblies from a range of *k*-mer values are merged, limiting the amount of sequence lost from the assembly. SPAdes also incorporates the BayesHammer short read correction tool (Nikolenko et al., 2013), which in combination with mapping reads back to the assembly to fix errors, can reduce the error profile in downstream applications. In addition, quality filtering to remove low quality regions from raw data has been demonstrated to improve the overall quality of genome assemblies (Del Fabbro et al., 2013).

Multiple bioinformatics pipelines have been published for *de novo* assembly, yet significant performance variation has been observed (Magoc et al., 2013). One of the most important discordances among assemblers is the amount of the assembly retained, based on benchmark comparisons using completed genomes. As sequencing platforms that generate longer reads become more widespread, completed bacterial genomes will continue to be automatically generated (Koren and Phillippy, 2015), removing the limitations when using incomplete draft assemblies. Until that time, short read assemblers should be chosen based on their completeness of draft assembly to reduce errors in SNP calling based on the presence or absence of homologous genomic regions.

***Calling SNPs using a genome assembly***

SNPs can be identified from genome assemblies, however, since coverage is 1x at each position in an assembly, spurious SNPs cannot be filtered due to insufficient coverage, nor can contaminating genomes be identified and subsequently removed. For individual genes, SNPs are identified by extracting alignments using BLASTN (Altschul et al., 1990) followed by pairwise alignment of the SNPs. For whole genome assemblies, SNPs are typically identified from whole genome alignments made with software such as MUMmer (Kurtz et al., 2004), Mugsy (Angiuoli and Salzberg, 2011), and Mauve (Darling et al., 2004). Software has also been developed for the identification of SNPs from genome assemblies for whole genome phylogenetics including kSNP (Gardner and Hall, 2013) and parSNP (Treangen et al., 2014). SNP identification using assemblies is useful when analyzing individual genes, processing huge datasets, or if raw reads are unavailable. However, when using assemblies for SNP discovery, SNPs cannot be evaluated and verified with the underlying raw read data.

## Sources of Error and Mitigation Strategies

**FIGURE 2:**
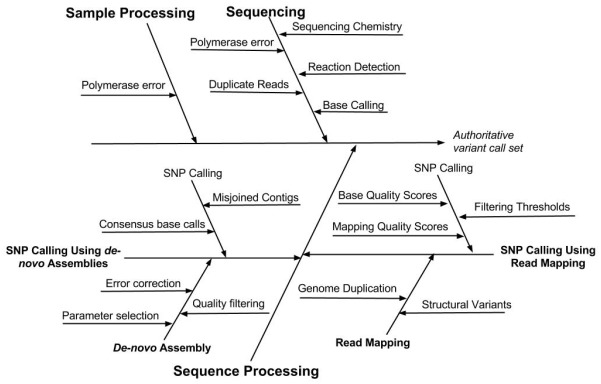
**Cause-effect diagram indicating the sources of error associated with different steps in the variant calling measurement process.** Note that the SNP calling is performed using one of two methods, either read mapping or *de novo* assembly.

Understanding the types and sources of error associated with a SNP and indel calling procedure will not only facilitate evaluation of results but also enable the user to optimize method performance. Several types of errors can impact the accuracy of SNP and indel variant identification. These errors occur during sample processing, the chemical and electronic processes that occur during sequencing, as well as the bioinformatic processing of sequence data: base calling, read mapping or *de novo* assembly, and identification of SNP and indel variants (Nielsen et al., 2011). The sources of error associated with different steps in the measurement process are depicted in Figure 2.

The errors occurring during the above processes can be random or systematic in nature. Random errors occur in an unpredictable manner, but given a large enough sample size may not lead to inaccurate results. Systematic errors occur in a predictable manner and if not accounted for, may lead to inaccurate results. Additionally, systematic errors, when unaccounted for, often result in a bias or a predictable difference between the true value and a measurement result.

The relative impact of the error can vary based on the sample preparation method, sequencing platform, or bioinformatic analyses used. The following section discusses the types of errors that may occur, along with possible mitigation strategies to minimize errors associated with FP or negative variant calls.

### Errors Associated With Sample Processing

As most NGS platforms require amplification of the genetic material, random and systematic sequence errors associated with sequence library preparation are primarily due to DNA polymerase infidelity during replication. These replication errors result in improper bases being inserted, deleted, or substituted at any given position (Ross et al., 2013). Substitution errors are random and subject to the fidelity of the DNA polymerases (1 error in 10^3–6^ bases, Showalter and Tsai, 2002), but can occur at a rate of up to 1,000–10,000 times lower than typical sequencing error (1 in 10^2–3^ bases McElroy et al., 2014). With sequencing depth commonly greater than 20X, random polymerase errors have minimal impact on variant calls (Cline et al., 1996; Metzker, 2010; Zook et al., 2012) and are usually only of concern when detecting minor component variants. Most current sequencing library preparation systems involve the use of high fidelity polymerases, further minimizing polymerase errors. However, homopolymers (sequence of identical bases, e.g., AAAA or TTTT) and tandem repeats (adjacent repeated patterns of two or more nucleotides) are known to experience higher replication indel error rates. Indel replication error rates, depending upon size and type of repeat array, can approach sequencing error rates (Vogler et al., 2006). Both substitution and indel polymerase errors accumulate linearly with increasing cycle number during amplification and then exponentially if erroneous amplification products become the primary template for subsequent cycles (PCR duplicates), at which point they may significantly contribute to variant call error (Kozarewa et al., 2009). Hence, minimizing PCR amplification, or foregoing it altogether, will minimize polymerase-introduced errors.

The use of paired-end library preparation methodology can also reduce variant calling error. Standard paired-end methodology (200–500 bp inserts between sequence reads) can help reduce errors during read mapping. Overlapping paired-end methodology (partial/complete overlap of forward and reverse reads from the same molecule) can provide a filter for removing random substitution error (i.e., only variants on both strands are called), thereby improving the ability to detect rare substitution variants by orders of magnitude (Schmitt et al., 2012; Chen-Harris et al., 2013; Colman et al., 2015). An even more recent advance is the “circle sequencing” approach, in which templates are circularized, copied via rolling circle amplification, and sequenced. This approach has led to exceptionally low error rates while maintaining relatively high sequencing yields (Lou et al., 2013).

### Errors Associated With Sequencing

Sequence generation errors are dependent on the sequencing platform and can be both random and systematic in nature, with the latter leading to local “hot spots” for high error rates (Ross et al., 2013). These have been found to be as high as 6% error for Illumina and 50% for Roche-454 (McElroy et al., 2014). The Illumina platform chemistry utilizes reversible dideoxy terminator nucleotides labeled with fluorescent dyes that have some degree of overlap in their excitation and emission spectra. While the overlapping excitation spectra allow for the use of only two lasers for all four nucleotides, the overlapping emission spectra, particularly for the G and T channels, and may result in base calling errors (Meacham et al., 2011). In fact, a number of systematic sequencing errors have been described for the various sequencing platforms (Ross et al., 2013). Some systematic sequencing errors have distinct characteristics, such as strand bias, which can be used to distinguish them from likely true variant calls. Other systematic errors are not well characterized, thus are more problematic, even with higher sequencing coverage (Shen et al., 2010), and warrant further investigation.

### Errors Associated With Sequence Processing

After the sequence data are generated, and as alluded to earlier, additional errors may occur during the mapping of the sequence reads to a reference genome or *de novo* assembly, and/or during variant calling.

#### SNP Calling Errors When Mapping Reads to a Reference

***Read mapping errors***

The two most common sources of true read mapping errors are genomic duplication and structural variation (Alkan et al., 2011). If multiple regions contain only slightly divergent sequences, then reads can potentially map incorrectly, leading to FP variant calls at those positions. Reads that map to “duplicated” regions in the reference genome are usually given a low mapping quality score by the algorithm, which are typically filtered. As previously mentioned, the use of paired-end libraries can reduce duplicate mapping errors if one member of the read pair maps to a unique location, thereby acting as an anchor for the proper mapping of the paired read to the correct duplicated region. Filtering out ambiguously mapped reads can mitigate FP variant calls due to mapping errors, but may also remove correctly mapped reads with true variants, potentially resulting in FN variant calls.

Alignment errors can also occur in regions containing small indels or structural variation if reads are mapped to the correct location but misaligned or allowed to extend into the structural variant, resulting in FP or FN variant calls (Subramanian et al., 2013). Optimizing the mapping criteria (e.g., *k*-mer size, number of ambiguous bases per *k*-mer allowed, etc.) for the organism under study can help mitigate the above errors. In general, highly diverse regions of the genome are more prone to mapping and alignment errors than lower diversity regions (Nielsen et al., 2011).

***SNP calling errors using mapped reads***

In an attempt to minimize variant calling errors, many variant calling algorithms calculate statistics such as strand bias, base quality rank sum, and neighboring base quality. In addition, Bayesian statistics may be used to incorporate the mapping quality scores assigned by the mapping algorithm (Li, 2011). These statistics can be used to filter or remove FP variants, as discussed below (McKenna et al., 2010; Meacham et al., 2011; Zook et al., 2014). However, base call and mapping quality scores are not always strongly associated with many systematic sequencing, local alignment, or mapping errors (Dohm et al., 2008).

To remove likely FP variants, many variant callers often use additional filters such as a minimum depth of coverage threshold, base call frequency (e.g., > 90% of calls at a position being identical), masking of homopolymer and repetitive/duplicated sequence regions, and trimming of poor quality bases from the ends of reads. These filters can be hard filters with carefully chosen cut-offs, or can be chosen by machine learning algorithms if sufficient training data are available (e.g., human genomes). One such algorithm is the GATK Variant Quality Score Recalibration (DePristo et al., 2011). It is important to recognize that real variants can be filtered out with FP, leading to higher FN rates. To reduce FN, manual inspection of reads mapped to filtered variants can be used to help identify true variants, thus reducing the number of FN variant calls (Zook et al., 2014). Also of note, strand bias can be defined in two ways: (1) a disproportionate number of reads from only one strand mapping to the reference or (2) a disproportionate number of reads with variant bases from only one strand. The second definition is particularly useful for identifying systematic sequencing errors caused by a particular motif (e.g., the GGT to GGG error with Illumina chemistries, which only occurs in reads in one sequencing direction; Meacham et al., 2011; Ross et al., 2013).

#### Variant Calling Errors When Using *de novo* Assemblies

***De novo assembly errors***

When using genome assemblies for variant detection, errors can be introduced in multiple ways, including the intrinsic error rate attributed to each sequencing platform. One approach to limit the effects of the errors inherent with short read chemistries is to use short read error correctors such as Musket (Bian et al., 2013) and Hammer (Medvedev et al., 2011) prior to genome assembly. Following assembly, these errors can be difficult or impossible to identify (Baker, 2012). After the genome is assembled, systematic errors can be corrected using bioinformatics tools such as the PAGIT pipeline (Swain et al., 2012). The recently published Pilon pipeline (Walker et al., 2014) can correct both SNPs and short insertions/deletions, and can also identify and fix incorrectly joined contigs. These best practices to reduce assembly errors can reduce their effect on downstream SNP applications.

***Variant calling from de novo assemblies***

Assembly errors are not the only form of error that can affect SNP calling applications using genome assemblies. For example, the whole genome alignment step can introduce errors. One of the most commonly used whole genome alignment methods is MUMmer (Delcher et al., 2002), which includes the nucmer program for aligning nucleotide sequences (Kurtz et al., 2004). By default, nucmer will align through large stretches of SNPs potentially introduced by misjoined sequence, or through artifact sequence introduced into the assembly (e.g., adapter sequence). Although these artifacts can typically be identified and removed, they can potentially introduce large numbers of erroneous variants into an analysis. Modifying nucmer parameters to specific datasets can help diminish the background noise introduced by these artifacts.

Another source of error for variant discovery in genome assemblies is the incorporation of homopolymer stretches, which are common to specific sequencing platforms (Loman et al., 2012). The incorporation of homopolymers into genome assemblies could generate incorrect variant calls, especially in the case of indels. Therefore, indels that are composed of homopolymers need to be verified with direct read mapping.

Additional errors may occur when calling variants that could be part of a mixture of multiple alleles. During assembly, a mixture of bases at a given position is collapsed down into a single base call. Although some of these errors can be corrected with methods discussed above, not all can be corrected and will serve as a source of error in downstream applications.

## General Guidelines for SNP Calling Method Evaluation

Defining analytical requirements is critical to method evaluation. The accuracy requirements of SNP and indel calling methods vary by application. For example, a small number of inaccurate SNP and indel calls may not alter phylogenetic inferences made for samples when a large number of SNPs and indels are available for analysis (Harris et al., 2013). However, for smaller total numbers of variants, individual SNPs and indels can have a greater impact on the phylogenetic interpretation. For example, for the investigation of the 2011 *E. coli* O104:H4 outbreak in Germany, only 19 SNPs were used to differentiate the isolates (Grad et al., 2012). Similarly, isolates from the *Bacillus anthracis* Ames strain and Haitian *Vibrio cholerae* outbreak investigations were based on relatively few SNPs (Hendriksen et al., 2011; Rasko et al., 2011), thus, accurate SNP calls were essential. Currently, the impact of individual variant calls on a phylogenetic analysis in relation to the total number of variants used to generate the analysis is unknown, but warrants further exploration.

A few general principles can help guide method evaluation. First, benchmarking datasets or samples used for evaluation must be critically evaluated with credible accuracy and be representative of the range of sample types used in the study or application (Ellison and Williams, 2012). Whenever possible, evaluation should be performed with data from well-characterized genomic DNA reference materials (RMs) or plasmid standards, if available and representative. Second, there must be one or more metrics for evaluating algorithm performance.

### Selection of Material for use in Benchmarking

Selection of appropriate samples, datasets, and reference genome is critical to robust characterization and evaluation of a variant calling measurement process.

#### Benchmarking Samples

The use of well-characterized genomic RMs allows for evaluation of the variant calling measurement process from sample processing through variant calling (Figure 1). Furthermore, to evaluate variant calling methods, the same data set must be processed by each of the different analysis pipelines being evaluated. The use of the same dataset is problematic because many variant callers are specific to a single sequencing platform or aligner, therefore evaluation of these variant callers using datasets generated by other platforms or aligners would lead to a biased representation of the caller’s performance. Therefore, the only way to perform complete and adequate comparisons is to use the same RM to generate data from different sequencing platforms and chemistries that the variant calling method will encounter.

A number of genomic RMs, along with respective reference sequence data, are available or are being developed. The genome sequences for currently available microbial genomic DNA RMs have not yet been rigorously characterized, to the authors’ knowledge. The National Institute of Standards and Technology (NIST) is currently developing whole genome microbial and human DNA NIST RMs (Zook et al., 2014^1^). These microbial RMs will provide valuable resources for challenging variant calling algorithms using well-characterized data. The microbial whole genome RMs are candidate NIST RMs 8375, 8376, 8377, and 8378. The RM strains were selected based on relevance to food safety and clinical settings and to represent a range in GC content. The four microbial genomes are *Salmonella enterica* subsp., *enterica* serovar Typhimurium LT2 (RM8375), a *Staphylococcus aureus* clinical isolate MRSA strain (RM8376), a *Pseudomonas aeruginosa* clinical isolate (RM8377), and a *Clostridium sporogenes* isolate (RM8378).

Another sample resource may be a set of plasmids containing known variants. The development of plasmid sequencing controls may be the best option for laboratory RM for rare variant detection. Through a collaboration, the Translational Genomics Research Institute (Flagstaff, Arizona) and Northern Arizona University (Flagstaff, Arizona) have developed a plasmid control using the pUC18 plasmid backbone, a widely used and commonly available cloning vector. DNA replication *in vivo* based on a plasmid represents the highest fidelity system for producing a sequence standard. The pUC18 plasmid is ∼2.7 Kbp, and DNA fragments containing SNPs or indels of interest can be readily inserted or deleted. By mixing different SNP- or indel-containing plasmids at known proportions, one can readily evaluate sequencing reads of these known mixtures. Another group successfully used plasmid controls to aid in the evaluation of rare variant detection (Cushing et al., 2013). An added benefit of plasmids as RMs is that their small size allows them be added into sequencing runs as internal controls alongside other samples. The error rate for plasmids within each sequencing run can then be directly measured. In fact, this is common practice with the phiX genome in Illumina sequencing runs.

#### Benchmarking Datasets

Sequencing datasets can be used to evaluate the variant calling measurement process, but do not aid in evaluation of sample processing and sequencing components of the measurement process (Figure 1).

***Real sequencing data***

Real data are the ideal source of sequence data for use in evaluating variant calling algorithms. Sequence data for the NIST microbial reference data are available through the NCBI sequence read archive (BioProject Accession PRJNA252728). Current efforts are underway to characterize the genome sequence of these RMs and will be made publicly available for use in variant calling method evaluation. While sequence data from well-characterized RMs are ideal, these data are not always available or representative of the intended use cases. When appropriate reference data are not available, alternative sources of sequence data are needed.

One such alternative source is sequence data from isolates sequenced on multiple sequencing platforms by multiple sequencing centers. Sequencing manufacturers commonly use *Escherichia coli* K-12 DH10B and MG1655 strains to benchmark new sequencing chemistries and provide this data on their web-sites. In addition, the Broad Institute (Cambridge, MA, USA), Joint Genome Institute (Walnut Creek, CA, USA), and J. Craig Venter Institute (Rockville, MD, USA) use *E. coli* K-12 MG1655 for quality control, and some of the quality control data are available through the GenBank Sequence Read Archive (Interactive web application for exploring datasets^2^). Multiple sequence datasets are also available (archived in GenBank SRA) for *Staphylococcus aureus* subsp. *aureus* TW20 and *Bacillus subtilis* strain 168. However, it is important to keep in mind that sequence variation may exist between stock cultures of the same strain at different sequencing centers. This is the primary advantage of RMs, as they are characterized for material homogeneity within a batch. Although a well-characterized genome sequence may not be currently available for these organisms, validated variant call sets can be generated using approaches described in the next section.

Once a dataset has been selected, there are several approaches for generating a genome sequence as the reference sequence data source. One approach is to combine sequence data sets from multiple sequencing platforms (Zook et al., 2014). Integrating data from multiple platforms has two key advantages: biases from each platform can be identified and down-weighted when integrating the data and variant callers can be tested on data from different sequencing platforms. Additionally, some laboratories have used SNP arrays to confirm SNP calls from NGS platforms (Goya et al., 2010; Subramanian et al., 2013). This approach works for already identified and more easily characterized SNPs, but is cost prohibitive for newly discovered SNPs. Furthermore, arrays do not always work when there are SNPs neighboring the variants of interest or in low quality mapping regions (Zook et al., 2014). With a characterized genome sequence, a known set of variant calls can be further validated through comparison of the characterized genome sequence to phylogenetically-related organisms or through the insertion of simulated variants.

***Simulated sequence data***

Simulated sequencing datasets can be used to validate variant calling methods when the available reference datasets do not adequately represent the variant calling methods. A primary advantage of using simulated data for method evaluation is that the true variants are known. Additionally, simulated datasets can be used in conjunction with reference datasets to test method robustness. Numerous sequencing read simulators are available, but differ in the error model (empirical or theoretical) used to generate sequencing datasets. Additionally, some read simulators do not introduce sequencing errors and thus can be used to define a baseline for algorithm performance in the absence of such errors.

Empirically-based read simulator algorithms vary in complexity. GenSim 1.0 (Engle and Burks, 1992) and MAQ (Gallagher and Desjardins, 2008) assume a uniform or constant error rate for all positions within a sequencing read. However, error rates are known to increase toward the ends of sequencing reads. Thus, more sophisticated error models have been developed to incorporate position-specific error rates (Engle and Burks, 1994; Richter et al., 2008; Holtgrewe, 2010; Jia et al., 2013). A number of read simulators use error models with sequence-specific error rates, for example homopolymers (Hu et al., 2012; McElroy et al., 2012). The benefit to this approach is that it more closely models sequence-specific errors in true sequencing datasets compared to models that utilize uniform or position-specific error rates. Quality scores from sequenced datasets can also be used to model error rates (Frampton and Houlston, 2012; Jia et al., 2013). Coverage bias and/or low coverage in GC-rich regions are also incorporated into some empirical error models (Frampton and Houlston, 2012; Hu et al., 2012). Some read simulators provide algorithms that allow the user to generate new error models, e.g., GemErr (McElroy et al., 2012; Jia et al., 2013). Whether a default error model is used or the user generates his/her own error model, a dataset with ground truth base calls is necessary in order to generate accurate error models, as inaccuracies in the reference genome would be incorporated as sequencing errors in the model.

Sequencing read simulators can also use theoretical error models based on the sequencing chemistry and sample preparation, and the reaction detection methods (Myers, 1999; Hazelhurst and Bergheim, 2003; Richter et al., 2008; Angly et al., 2012^3^). The advantage of theoretical models is that they are able to incorporate sources of error that are difficult to include in empirical models.

In addition to modeling base call error rates, it is also important to model BQS. However, some read simulators do not provide quality scores, e.g., metaSim (Richter et al., 2008), celsim (Myers, 1999), and GenSim (Engle and Burks, 1992). Alternatively, fixed quality values are generated by Grinder (Angly et al., 2012). Correct bases are assigned a quality score of 30 and error positions are assigned a score of 10. The pIRS by Hu et al. (2012) uses quality scores from an existing sequence dataset. While this approach is more representative of the distribution of quality scores in a sequencing dataset, it assumes the quality scores are accurate.

The assigned BQS can also reflect the model uncertainty. For example, flowsim (Balzer et al., 2010) uses Bayes theorem to assign quality values for simulated base calls. Huang et al. (2011) takes the opposite approach, modeling quality scores and using those assigned quality scores for the base call error model. Using an empirical approach to simulate BQS values, Li et al. (2008) generated a position-specific quality score model for error and non-error bases by modeling the quality score distribution using a first order Markov chain. To fully evaluate SNP calling algorithms using simulated sequence data, both sequences and quality scores must simulate real sequence data as accurately as possible.

Finally, when using simulated datasets, a random number seed value must be defined. Random numbers are used during the generation of simulated reads and they require a seed number. This seed number is used to produce the set of random numbers. Reusing the same seed number results in the production of the same set of random numbers. To enable reproducibility in the SNP calling algorithm evaluation procedure, the user should rerun the simulation using the same seed value, however, different seed numbers should be used when generating replicate datasets.

#### Reference Genome Selection

The choice of the reference genome for SNP calling can bias which SNPs are called, e.g., SNPs in genes not in the reference genome will not be called, and these effects can be observed after phylogenetic tree construction. This potential bias has less of an effect in clonal bacteria, in which there are few genomic variants among the clones, even when the clones are compared to different species (Foster et al., 2009; Pearson et al., 2009). When working with genetically diverse genomes, using multiple references (Bertels et al., 2014) better reflects the diversity in the number of SNPs and genomic complexity (e.g., repeat regions and structural variants) the algorithm will face. While accurate phylogenetic reconstruction of genetically diverse strains requires using multiple reference genomes representing a range of genomic similarity to the strains being compared, the same biases and challenges exist for SNP calling. In fact, SNP calling can be conducted against multiple references during validation of the SNP discovery process (Shen et al., 2010; Pightling et al., 2014).

Just as simulated whole genome sequence data can be used to evaluate algorithm performance, reference genomes with simulated mutations can also be used. The benefit to using reference genomes with simulated mutations is that they can provide a ground truth while using actual sequencing reads. Reference sequence mutation algorithms can simulate substitutions, as well as insertions and deletions. Some examples of reference sequence modification tools able to introduce SNPs into a reference sequence include: fakemut, part of the Maq sequence mapping algorithm tool (Gallagher and Desjardins, 2008), GemHap from GemSim (McElroy et al., 2012), and mutatrix^4^.

### Performance Metrics

A variety of performance metrics have been derived and/or borrowed from other disciplines, including calculations of true and FP and negative call rates, as well as more sophisticated calculations that attempt to summarize algorithm performance in a single value (Table 1). Graphical representation of these metrics, such as precision versus recall plots and Receiver Operating Characteristic (ROC) curves, can be very useful in visually depicting the different performance aspects of various algorithms. This section discusses common performance metrics, use of replicates for determining metric uncertainty, as well as data visualization approaches for interpreting performance metrics.

**Table 1:** **Definitions of common performance metrics used in evaluating variant callers**.

**Metric**	**Calculation**	**Interpretation (Ideal)**	**Alternative names**
Accuracy	$\frac{\text{TP+TN}}{\text{TP+FP+TN+FN}}$	Ratio of correct calls to total calls and variants (1)	
Specificity	$\frac{\text{TN}}{\text{TN+FP}}$	Non-variants not called as variants relative to the total non-variants (1)	
Sensitivity	$\frac{\text{TP}}{\text{TP+FN}}$	True variants called relative to all variants (1)	Recall, true positive rate (TPR), positive call rate
Precision	$\frac{\text{TP}}{\text{TP+FP}}$	True variants called relative to total calls (1)	Positive predictive value (PPV)
False positive rate	$\frac{\text{FP}}{\text{TN+FP}}$	Non-variants called relative to the total non-variants (0)	

#### Selection and Calculation of Performance Metrics

**FIGURE 3:**
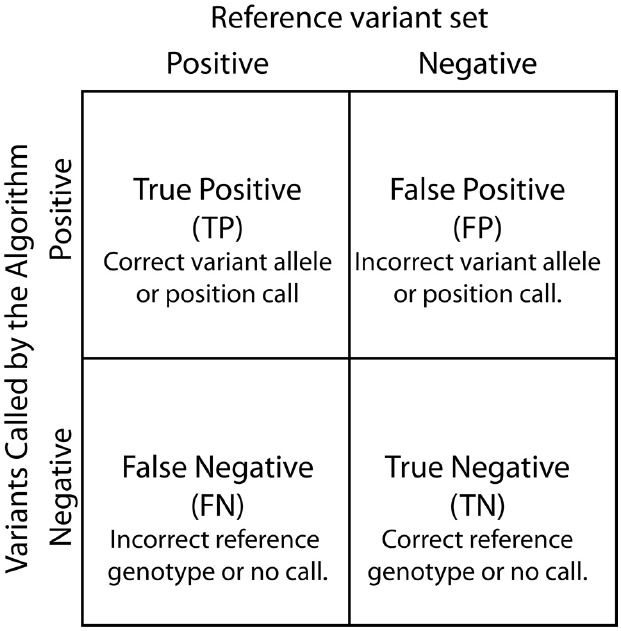
**Contingency table.** True Positive, False Positive, False Negative, and True Negatives are defined based by the relationship between variants called by the SNP calling algorithm and known differences between the reference genome and the analyzed sample.

Contingency tables, also referred to as confusion matrices, are used to evaluate classifiers such as variant calling algorithms (Figure 3). Two by two contingency tables present the relationship between variant labels assigned by an algorithm and labels from a declared truth set. The four basic values in a 2 × 2 contingency table: true positive (TP), true negative (TN), false positive (FP), and false negative (FN), are used to assess algorithm performance. Of note, contingency tables can be functions of a parameter value or threshold choices. For example, changing the variant call quality score threshold used to determine variant call sets can alter the resulting contingency table.

Several performance metrics have been developed to characterize classifiers using contingency tables (Baldi et al., 2000). Here, we discuss five of the most commonly used metrics (Table 1). Accuracy is a single numeric summary of the four values representing the ratio of the correct calls and non-calls to the total number of variant and non-variant positions. The other four metrics can be interpreted as conditional probability statements relating the algorithm’s variant calls to a reference variant call set. Specificity is the probability that a reference non-variant is called as non-variant. Sensitivity is the probability that a reference variant is called as a variant. Precision estimates the probability that a variant call is truly a reference variant, whereas the FP rate is the probability that a variant call is falsely called in the sample.

Determining which metric to use is up to the user; however, the authors suggest some or all of the commonly used metrics presented here should be included when evaluating algorithms. When determining which performance metrics to use, one should consider the assumptions and requirements for the performance metric (Table 1). For example, some metrics depend on the proportions of positive and negative variants in the benchmark dataset, whereas sensitivity and specificity are independent of these proportions. Another consideration for selecting performance metrics is whether the purity or completeness of a call set is more desirable. For example, strict filtering criteria are used to identify high predominately TP variant calls, however this may leave the set incomplete and potentially misrepresentative of the population of variants. Accuracy and precision provide insight into completeness, whereas sensitivity and specificity measure completeness. In the end no matter what metric is used, it must be properly interpreted based on the application or context in which it is applied.

When comparing performance metrics across different variant calling algorithms, it is important to consider each metric’s associated uncertainty. The performance metric uncertainty applied to variant calls from data may be quantified using a bootstrapping protocol. For simulated datasets, repeated simulations can be used to calculate performance metric uncertainty. For example, Zeng et al. (2013) performed 10 replicate simulations and compared sensitivity, specificity, and F-score [2 × (precision × recall)/(precision + recall)] for four variant callers at three different coverage values (5X, 10X, 15X). The desired performance metrics were then calculated for each dataset and the resulting values were used to calculate the performance metric uncertainty. Replicate simulations can be generated for either the sample dataset or reference sequence. If the sample dataset is replicated, the variant calling algorithm performance can be evaluated for specific variant locations as well as for overall performance. However, as variant detection can be sequence context-dependent, using only one set of simulated variants may lead to bias in favor of variant calling algorithms better suited for the sequence context of that variant call set.

#### Resources for Comparing and Classifying Variant Calls

A variety of tools have been developed for assessing performance of variant calls with respect to benchmark variant calls for a sample. Currently, these tools are mostly command line based and optimized for human genomes, but many can be applied to microbial genomes as well. The bcbio.variation tool^5^ can regularize vcf files, compare them to a benchmark call set, and generate a variety of metrics, including sensitivity, specificity, and genotyping error rate. The SMaSH benchmarking toolkit (Talwalkar et al., 2013) can generate metrics for precision and recall of mappers and variant callers. A novel aspect of SMaSH is the calculation of uncertainty of precision and recall due to imperfect benchmark call sets. Another tool, the vcflib library^6^, can normalize many complex variants, generate ROC curves, and perform comparisons across vcf files. The USeq VcfComparator tool^7^ can generate ROC curves and compare only variants inside bed files. GATK also has tools to combine and compare variant and genotype calls (McKenna et al., 2010; DePristo et al., 2011; Van der Auwera et al., 2012). Finally, the vcfeval tool in RTGtools can compare a vcf file to a benchmark dataset and produce ROC curves and lists of TP, FP, and FN variants (RTG paper under review^8^). Two other new tools being developed by members of the Global Alliance for Genomics and Health Benchmarking Team are hap.py^9^ and vgraph^10^, both of which are able to compare complex variants using graph representations. Many of these tools are being actively developed and their application to microbial systems presents an opportunity for future research.

Finally, when a well-characterized set of variant calls is not available for benchmarking, latent mixture models can be used to predict rates for the truth table values (Kim and Speed, 2013; Cantarel et al., 2014). Latent mixture models predict the model of the underlying data (true variant call set) based on the responses from multiple variant calling algorithms. Latent mixture models treat the true mutation status as the latent or unknown variable. The model can then be used to estimate the FP and FN rates from which the TP and TN rates are calculated. While this method has been frequently used to validate biomedical assays, evaluation of algorithm performance relative to a known truth is preferred, as the latent mixture model may be susceptible to an unknown bias.

#### Visual Comparison of Performance Metrics

Graphical presentation of performance metrics can facilitate the comparison and evaluation of algorithm performance. The appropriate data visualization method depends on the discreteness or continuity of the variables involved. For variant algorithm evaluation, a single set of variants generated by an algorithm provides an example of a discrete variable, and the quality value assigned to each candidate variant provides an example of a continuous variable. We analyzed 16 replicate datasets using two different methods (A and B), generating 32 sets of variant calls. The variant call sets were then used to demonstrate different methods for visually comparing variant calling algorithms and their performance metrics discussed in the previous section. The R code and input data used to generate the figures are available at https://github.com/nate-d-olson/snp_pipeline_evaluation.

**FIGURE 4:**
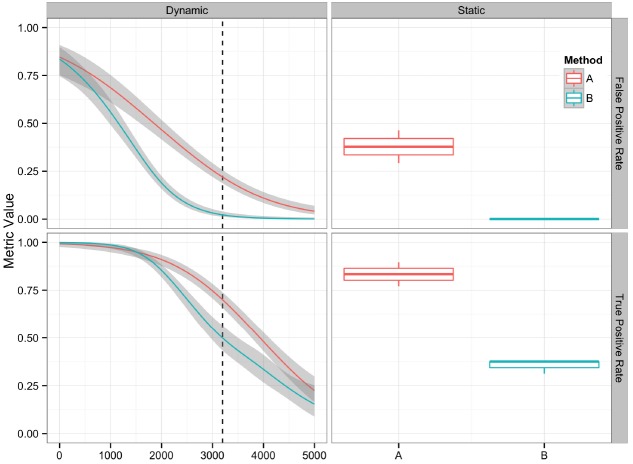
**Comparison of two variant calling algorithms using two of the performance metrics in Table 1, true positive rate and false positive rate, calculated from contingency tables over a range of quality score thresholds (left) and a fixed quality score threshold (right).** Methods A (red) and B (teal) indicate two different variant calling methods. Left: A smoothing function (generalized additive model) was used to summarize the contingency table metrics across the considered quality score interval. Red and teal lines are smoothing functions, and the gray area represents the 95% confidence interval. The vertical dashed line indicates the quality score cutoff (*Q* = 3195) for the static tables. Right: Boxplots are used to summarize the performance metrics calculated from the contingency table value for the replicate datasets at the defined cutoff value.

**FIGURE 5:**
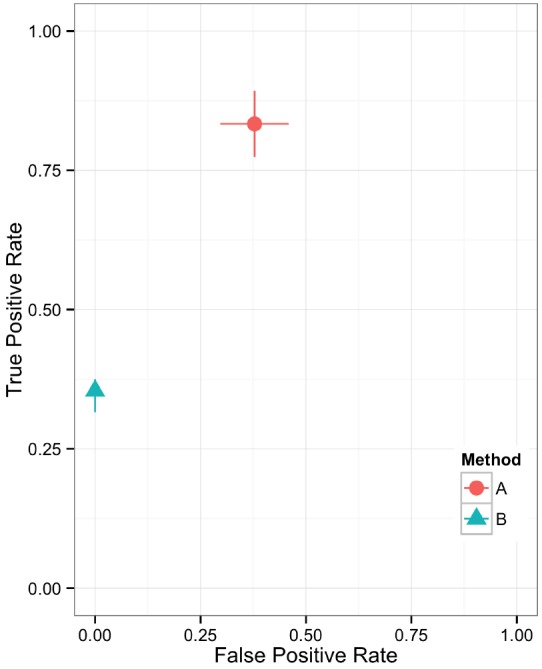
**Scatter plot showing the relationship between two performance metrics for variant call sets.** The individual data points are based on metrics calculated from static contingency tables. The error bars represent the 95% confidence interval for each performance metric.

**FIGURE 6:**
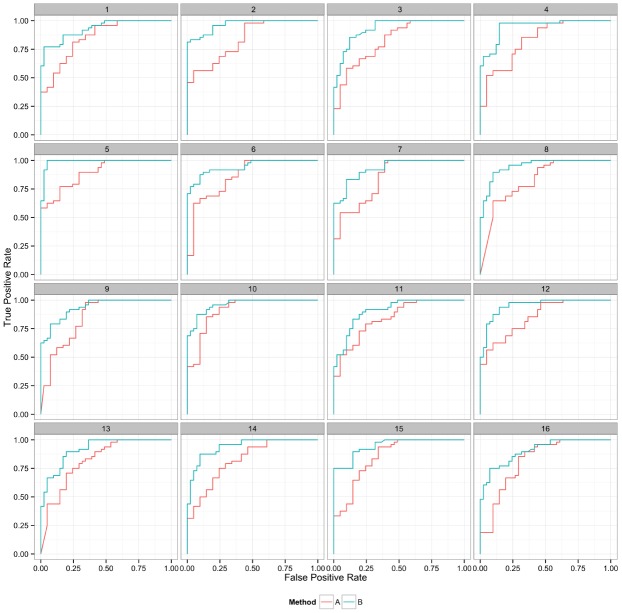
**Curves represent the relationship between the two performance metrics, in this case true positive and false positive rate.** Replicate samples are plotted individually to indicate the robustness in the relative performance of the two methods.

Plotting the value of a performance metric as a function of the chosen threshold value for the continuous variable can be useful when comparing algorithm performance over a range of cutoff values. Figure 4 depicts the relationship between algorithm performance and variant quality values. The boxplots show how method A has higher FP and TP rates compared to B for a single threshold value. The dynamic plot with the smoothed data shows the range of threshold values for which this relationship holds. The range of threshold values for which a trend (e.g., method A has a higher FP rate compared to method B) is observed is an indication of the robustness of the trend. The ideal values for FP and TP rates are 0 and 1 respectively. As method A has higher values for both metrics compared to method B, a trade-off between accepting FP vs. FN will determine which method to use. Visualizing two metrics plotted against one another can more clearly present the trade-off between the methods. To compare method performance for two metrics with a fixed threshold, a scatter plot can be used (Figure 5). When considering a range of possible thresholds, one metric can be plotted as a function of a second metric (Figure 6). A ROC curve is an example of a comparison between two performance metrics over a range of threshold values. Values of the threshold or additional metrics can be indicated through variations in symbol or line color, width, or pattern. When many metrics are presented, it may prove most effective to plot each metric against a common variable, such as threshold. The uncertainty of the metrics can be presented in a number of ways. Here the uncertainty is represented qualitatively through the comparison of the two methods relative performance across a collection of 16 replicates. The uncertainty of the metrics can also be presented as error bars representing the uncertainty of one metrics given a fixed value for the second metric (marginal distributions), or the combined distribution of both metrics (joint distribution).

While ideal for many purposes, available reference variant call sets may not be appropriate for a desired application. When an appropriate benchmark variant call set is not available, Venn diagrams can be use to compare variants called using different methods. However, use of Venn diagrams for method comparison should be supplemented with simulated data for the target application. It is important to note, however, that variants called by multiple algorithms are not necessarily true variants, as multiple variant callers can be susceptible to the same biases.

## Conclusions

When using genomic variants for phylogenetic analysis, comparative genomics, or outbreak investigations, it is critical to properly evaluate the variant calling method. Many sources of error are associated with sequencing as well as variant calling. To optimize the quality of the data used to generate the variant calls, it is advised to minimize amplification during sequencing library preparation, perform paired-end sequencing, remove duplicate reads, realignment around indels, and perform BQS recalibration.

A thorough evaluation of variant calling methods would include: (1) The use of multiple datasets with known authoritative variant call sets that represent the range of data the algorithm will evaluate. The datasets used for evaluation could be real, simulated, or a combination of both; (2) Replicates of different sequence datasets or reference genomes to calculate performance metric confidence intervals; (3) Performance metrics that aid in evaluating algorithm performance. With method evaluation performed in this manner, the user will be able to understand positives and negatives of algorithms for the application of interest and characterize the level of confidence in variant calls. We are currently working to follow this review with the development of complementary open source tools for use in evaluating variant calling algorithm performance, with a focus on calculating and presenting performance metrics.

### Conflict of Interest Statement

Opinions expressed in this paper are the authors’ and do not necessarily reflect the policies and views of the Department of Homeland and Security (DHS), National Institute of Standards and Technology (NIST), or affiliated venues. Certain commercial equipment, instruments, or materials are identified in this paper only to specify the experimental procedure adequately. Such identification is not intended to imply recommendation or endorsement by the NIST, nor is it intended to imply that the materials or equipment identified are necessarily the best available for the purpose. Official contribution of NIST; not subject to copyrights in USA.

## Footnotes

1 http://genomeinabottle.org

2 https://github.com/nate-d-olson/snp_eval_shiny

3 http://www.ebi.ac.uk/goldman-srv/simNGS/

4 https://github.com/ekg/mutatrix

5 https://github.com/chapmanb/bcbio.variation

6 https://github.com/ekg/vcflib

7 http://useq.sourceforge.net/

8 http://realtimegenomics.com/products/rtg-tools/

9 https://github.com/sequencing/hap.py

10 https://github.com/bioinformed/vgraph
